# Terpene Synthase Genes in *Quercus robur* – Gene Characterization, Expression and Resulting Terpenes Due to Cockchafer Feeding

**DOI:** 10.3389/fpls.2018.01753

**Published:** 2018-11-30

**Authors:** Friederike Carolin Creyaufmüller, Isabelle Chassignet, Horst Delb, Aikaterini Dounavi, Oliver Gailing, Ludger Leinemann, Jürgen Kreuzwieser, Julia Teply-Szymanski, Barbara Vornam

**Affiliations:** ^1^Chair of Tree Physiology, Institute of Forest Science, University of Freiburg, Freiburg, Germany; ^2^Department of Forest Protection, Forest Research Institute Baden-Württemberg, Freiburg, Germany; ^3^Department of Forest Genetics and Forest Tree Breeding, University of Göttingen, Göttingen, Germany

**Keywords:** root volatile, terpene synthase (TPS) gene, root herbivore, oak provenance, climate change

## Abstract

Root herbivory caused by larvae of the forest cockchafer (*Melolontha hippocastani*) enhances the impact of drought on trees, particularly in oak forest rejuvenations. In Germany, geographically distant oak stands show differences in infestation strength by the forest cockchafer. While in Southwestern Germany this insect causes severe damage, oak forests in northern Germany are rarely infested. It is known that root-released volatile organic compounds (VOCs) are perceived by soil herbivores, thus guiding the larvae toward the host roots. In this work, we exposed seedlings of two distant oak provenances to forest cockchafer larvae and studied their population genetic properties, their root-based VOC chemotypes, their attraction for larvae and terpene synthase gene expression. Based on nuclear and chloroplast marker analysis, we found both oak populations to be genetically highly variable while showing typical patterns of migration from different refugial regions. However, no clear association between genetic constitution of the different provenances and the abundance of cockchafer populations on site was observed. In contrast to observations in the field, bioassays revealed a preference of the larvae for the northeastern oak provenance. The behavior of larvae was most likely related to root-released volatile terpenes and benzenoids since their composition and quantity differed between oak populations. We assume repellent effects of these compounds because the populations attractive to insects showed low abundance of these compounds. Five different oak terpene synthase (TPS) genes were identified at the genomic level which can be responsible for biosynthesis of the released terpenes. TPS gene expression patterns in response to larval feeding revealed geographic variation rather than genotypic variation. Our results support the assumption that root-released VOC are influencing the perception of roots by herbivores.

## Introduction

Global climate change will most likely lead to higher mean annual air temperatures as well as seasonal and spatial changes of precipitation patterns (Forster et al., 2007; Spellmann et al., 2007; IPCC, 2014). In Central Europe, these changes will result in considerably reduced summer precipitation and, therefore, in a higher probability of drought stress in forests. It is assumed that drought periods will at least partially be accompanied by heat waves. In addition to such expected more frequent and severe abiotic stress events, which weaken the vitality of trees, there might be an enhanced risk for the occurrence of biotic stressors. For example, there are hints that climate change will cause an increased abundance of certain insect herbivores (Bolte et al., 2009; Delb, 2013). One of these insects is the forest cockchafer (*Melolontha hippocastani* Fabr.). The adult beetles of this species are consumers of tree and shrub leaves and are potentially able to migrate long distances while the larvae or “grubs” can move horizontally in the soil within a range of 1.5–5.5 m (Hasler, 1986; Weissteiner et al., 2012). Larvae have been reported to cause severe root damage to a range of economically important trees and in particular to oak (Delb, 2004; Huiting et al., 2006); it is assumed that due to global warming the reproduction cycle of this insect species will decrease from 5–6 years to only 3–4 years (Delb, 2013; Kolář et al., 2013), which will further enhance the frequency of biotic stress for their host tree species in the future.

In Central Europe, the oak species *Quercus robur* and *Q. petraea* belong to the main forest trees with enormous economic and ecological value. These oak species show a wide geographic distribution in Europe as well as in Germany and they are dominant tree species in many forests growing under a broad range of climatic and edaphic conditions (Gailing, 2008). *Q. robur* is the most widespread oak species in Europe and it preferentially grows on clay or acidic, deep and well-watered soils. Moreover, it is resistant to strong winter frosts and tolerant to high summer temperatures and high light exposure (Jones, 1959). Since the studies of Petit et al. (2002) with maternally inherited chloroplast DNA markers much is known about the genetic differentiation of oaks in Europe as a result of the recolonization from different glacial refugia. Further investigations (Gailing et al., 2003, 2007; König and Stauber, 2004; Liesbach et al., 2006; Neophytou et al., 2010, 2015; Hosius et al., 2012) draw a very detailed picture of genetic variation and differentiation of oak stands in Germany. These investigations underline the function of oak species as an excellent model species to study the adaptation of forest trees to changing environments (Gailing et al., 2009). However, these findings also highlight that the genetic background of oak populations has to be considered in field studies when interpreting their stress tolerance.

It is well known that aboveground plant parts emit a large variety of volatile organic compounds (VOCs) into the atmosphere. Terpenoids, including monoterpenes and sesquiterpenes, represent the largest and most diverse class of volatiles released by plants (Dudareva et al., 2006). These compounds exert multiple functions in the interaction of plants with their environment. For example, they serve as attractants for pollinators and fruit dispersers or play a role as repellents in the defense against herbivores and pathogens (Huang et al., 2010; Irmisch et al., 2014). The biosynthesis of terpenoids is mediated either by the chloroplastic MEP pathway or the cytosolic mevalonate pathway (Dudareva et al., 2006). These biochemical routes provide the C_5_ compounds isopentenyl diphosphate and dimethylallyl diphosphate, which are used to synthesize the larger terpenes including monoterpenes (C_10_) and sesquiterpenes (C_15_). The final structure of terpenes is eventually formed by terpene synthases (Degenhardt et al., 2009).

Compared to aboveground plant parts, much less is known on the function of root-released VOCs. Nevertheless, specific information can be transferred over long distances in the soil by root-derived VOCs. For example, several studies indicate that they seem to be of significance for plant–fungi (Ditengou et al., 2015), plant–bacteria (Del Giudice et al., 2008) and plant–animal communication (Rasmann et al., 2005; Johnson and Gregory, 2006; Johnson and Nielsen, 2012). For example, Weissteiner et al. (2012) showed that root volatiles can be perceived by the olfactory system of cockchafer larvae and that this signal might be used by the animals for host plant location. Similarly, also other soil-inhabiting insects use root-released volatiles as a signal to orient themselves toward this food source (Nordenhem and Nordlander, 1994; Thomas et al., 2008). Interestingly, the blend of volatiles released by plant parts can change in response to herbivore attack because herbivore-induced plant volatiles (HIPVs) are synthesized (Robert et al., 2012; War et al., 2012). HIPVs are thought to be part of the plant’s defense arsenal against plant feeding herbivores. Nevertheless, it has been demonstrated that soil-dwelling larvae were especially attracted to roots of plants which were infested with conspecifics (Robert et al., 2012).

In the present study we aimed to describe the interaction of *Q. robur* populations with *M. hippocastani* based on the release of VOCs from plant roots. Several studies have demonstrated that distant populations or “provenances” of a given tree species form specific blends of terpenes which can be released into the environment (Staudt et al., 2008; Loreto et al., 2009; Welter et al., 2012; Kleiber et al., 2017). However, a clear correlation between provenances of a species and terpene chemotypes might be blurred by underlying evolutionary forces like paleographic fragmentation or hybridization and introgression events (Staudt et al., 2008; Loreto et al., 2009; Welter et al., 2012). Still, intraspecific variability of blends of volatile terpenes (i.e., chemotypes) in oaks has rarely been studied (Staudt et al., 2001, 2008; Loreto et al., 2009; Welter et al., 2012). Interestingly, investigations between geographically distant oak stands in Germany have revealed clear differences in the extent of infestation by the forest cockchafer. While oak stands in Northeast Germany are rarely infested with cockchafer larvae (Subklew, 1937), comparable stands in Southwest Germany regularly experience severe damage by larval feeding, which can even put the survival of oak progeny into danger (Delb, 2004). In spite of that, some stands in close vicinity to severely damaged stands were observed to be far less affected. In the present work, we therefore characterized the genetic structure of *Q. robur* populations in Germany that showed different degrees of cockchafer infestation.

We tested the hypotheses (i) that different *Q. robur* populations from distant locations in Germany are genetically differentiated and that genetic differentiation is associated with infestation strength, and (ii) that these populations form distinct VOC chemotypes. We further postulate (iii) that the attraction of cockchafer larvae is different among these oak populations and (iv) that it is associated with the release of specific terpenes and with the expression of terpene synthase genes. We also assume that (vi) terpene synthase single nucleotide polymorphisms (SNPs) differentiate between oak populations with high and low cockchafer population density. Furthermore, we hypothesize that oak populations in northern Germany have evolved no tolerance to cockchafer feeding, since the insect has been absent from this region. Therefore, the response of such oak populations to herbivory should differ from populations regularly attacked by cockchafer larvae. For this purpose, we quantified the composition of volatile blends in response to cockchafer feeding and studied the expression of terpene synthase genes in roots of the oak trees.

## Materials and Methods

### Site Characteristics and Plant Material

We selected tree individuals from a total of 19 pure and mixed oak stands in Germany (Supplementary Table S1). For population genotyping, leaf tissue was randomly sampled from at least 50 individuals per stand, either on site or directly from trees used in the experiments. For the experiments under controlled conditions, 3- to 5-year-old pedunculate oak (*Quercus robur* L.) seedlings of four populations were used. Two populations (“R36,” “R45”) originated from the Upper Rhine valley in Southwest Germany; two others (“BB1,” “BB2”) from the Federal State of Brandenburg in Northeast Germany. Either, they were grown in the greenhouse from seeds collected from a range of mother trees randomly distributed over the respective populations (SW German stands), or they originated from a local tree nursery (NE German stands). Consequently, none of the seedlings experienced cockchafer interaction prior to experiments and hence we could rule out any priming effects.

The major difference between those oak-dominated stands is their infestation strength. To the best of our knowledge, “BB1” and “BB2” have never experienced any infestation by the forest cockchafer, while population density is high in “R36” and low in “R45.” Cockchafer infestation strength in the Baden-Württemberg stands was inferred from a long-term monitoring program which assesses cockchafer population density at an areal level (Delb and Mattes, 2001).

### Insects

For the experiments, some 100 third instar larvae (“white grubs”) of the forest cockchafer (*Melolontha hippocastani*) were used. The animals were collected from a field site in the Upper Rhine Valley (48°49′36.3″N 8°10′30.5″E) by digging to a depth up to 60 cm. The grubs were stored in natural soil from the field site at 8°C for a maximum of 1 week until they were used in the experiments.

### Experimental Setups

#### Bioassays

To test if forest cockchafer grubs are particularly attracted by roots of specific *Q. robur* provenances, oaks of three provenances (“R36,” “R45,” “BB2”) were tested against each other. Plants were grown for 3 months in 50 L plastic pots (diameter 52 cm)^1^ with burnt quartz sand (Ø = 0.07–0.2 mm; 0.2–0.7 mm and 1.2–2 mm in relation 8:10:1) as a substrate, supplied with 4.5 g l^-1^ long term NPK fertilizer (Manna Cote 8M; Manna; Ammerbuch-Pfäffingen; Germany). The seedlings were grown under long day conditions (16 h light/8 h dark, PAR: 250–300 μmol m^-2^ s^-1^). They were watered daily with tap water. Four trees (two per provenance) were planted crosswise into the pots, placing trees of different origins opposite to each other (Supplementary Figure S1). Each of the three possible combinations was replicated five times resulting in a total of 15 pots. For the bioassays, self-constructed glass tubing systems (inner diameter 45 mm, length of each tube 100 mm) were dug 20 cm deep into the center of each pot. The tubing system was equipped with (i) an opening in the center facing upward for entering the larvae and (ii) four exits facing the root systems of the four oak seedlings. The whole glass tubing system was filled with the same quartz sand substrate as the pots. To increase the possible number of independent replicates, two opposing exits were closed with an air-impermeable film for the first set of experiments; for a second set of bioassay experiments, the impermeable film was shifted to the remaining two opposing exits. The two open exits were closed with a net to hinder grubs from escaping the glass tubing system. For starting the bioassays, one grub was placed carefully into the middle entrance of the system. After a 12 h period the glass tubing system was removed from the soil and checked for the location of the grub. Each grub was only used once. In the course of the experiment, the root-released VOCs were collected in 10 cm depth in the rhizosphere of each single tree with passive samplers as described below. After each experiment, the substrate was completely removed from the tubing system, replaced by new substrate and placed again into the center of the pot.

#### Herbivory Effects

To study the effects of herbivory on the gene expression of terpene synthases and on the release of VOC by oak roots, a “feeding experiment” using populations “R45” and “BB1” was conducted. For this purpose, 12 seedlings per population were individually planted into 5.5 L plastic pots (AgrarPeter, Teningen, Germany) and grown for 8 months in the same substrate and under the same conditions as given above for the bioassays.

For the experiment, two forest cockchafer grubs were transferred to each pot. For each of the three provenances six pots were infested with grubs (*n* = 6) and six trees of each provenance were kept uninfested as controls. At the same time, we placed three passive samplers (details see below) for adsorption of root-released VOCs in 10 cm depth in each pot. After 8 days of infestation, the passive samplers were collected, carefully cleaned from soil particles and stored at 8°C until analysis of VOCs which was done within 1–2 days after collection.

### Population Genetic Analysis: Microsatellite Genotyping With Chloroplast and Nuclear Single Sequence Repeats (cpSSR, nSSR)

In order to comprehensively characterize the genetic background of the plant material used for this study, we characterized the tree individuals from the different field sites in Germany at species-discriminating nuclear SSRs (Neophytou et al., 2010). Specifically, we used these markers for species assignments to make sure that only *Q. robur* trees were included in our experiments and to test whether genetic differentiation is associated with differences in infestation strength.

For population genotyping leave tissue was randomly sampled from at least 50 individuals for each of the 19 stands (Supplementary Table S1), either on site or directly from trees used in the experiments. Total DNA was extracted using the DNeasy 96 Plant Kit (Qiagen, Hilden, Germany). All samples were genotyped in six multiplexed PCR reactions amplifying the 11 nuclear SSRs QpZAG1/5, QpZAG9, QrZAG11, QpZAG16, QpZAG15, QrZAG30, QrZAG87, QrZAG96, QrZAG101, QpZAG110, QrZAG112 (Steinkellner et al., 1997; Kampfer et al., 1998) and the 10 chloroplast SSRs μkk4, μkk3, μcd5, μcd4, μdt4, μdt3, μdt1, ccmp10, ccmp6, ccmp2 (Weising and Gardner, 1999; Deguilloux et al., 2003) following the protocol of Neophytou et al. (2010). Capillary electrophoresis was performed in an ABI-PRISM-3130 Genetic Analyzer (Applied Biosystems) and the GeneMapper 4.0 Software (Applied Biosystems) was used for allele calling.

Population genetic analysis was performed in order to test for an association between genetic differences and differences in cockchafer abundance between populations. Each individual was assigned to one of the three oak species *Q. robur, Q. petraea* and *Q. pubescens* by cluster analysis in STRUCTURE v2.3.4 (Pritchard et al., 2000; Falush et al., 2003). Species-discriminant markers were used according to Neophytou et al. (2010) and four known pure stands (R36, V, F) served as reference. Only pure-bred *Q. robur* individuals (*q* ≥ 0.875) were chosen for further experiments, resulting in 14 pure *Q. robur* stands consisting of more than 10 individuals. Genetic variation within and among the resulting *Q. robur* populations was calculated using the software GenAlEx 6.5 (Peakall and Smouse, 2006, 2012). The Bayesian approach in STRUCTURE was used to reveal genetic structures corresponding to provenances or cockchafer population density. The admixture model was applied and analysis parameters were set to 100,000 runs in the burn-in period, followed by 100,000 MCMC repetitions. *K* was set to 1–20 with 10 iterations per *K*. The highest ΔK was calculated based on the rate of change between successive *K* values (Evanno et al., 2005). Chlorotypes were defined according to Neophytou and Michiels (2013). The refugial ancestry of the original seed material was inferred by comparison with the corresponding refugial linages (Petit et al., 2002). Chlorotype frequency within and genetic variation among all populations were calculated in GenAlEx.

### Identification of Genomic Terpene Synthase Genes by PCR

The orthologous sequences of several terpene synthase genes of *Populus trichocarpa* available in the EMBL database were used to find corresponding sequences by a BLAST search in the *Q. robur* transcriptome database publicly available at https://urgi.versailles.inra.fr/blast/blast.php and with a BLASTx search of the translated nucleotide sequences within the NCBI BLAST+ database (Supplementary Table S2). Primers (Supplementary Table S3) were designed by using the program Primer 3 (Koressaar and Remm, 2007; Untergasser et al., 2012; from S. Rozen & Whitehead Institute/MIT Center for Genome Research)^2^. Primers were quality checked using the program Oligo calc: Oligonucleotide Properties Calculator^3^. PCR amplification was performed in a 25 μl volume containing 10 ng template DNA, 10 mM Tris/HCl pH 9.0, 0.2 mM of each dNTP, 1.5 mM MgCl_2_, 50 mM KCl, 0.2 mM each of forward and reverse primer and 1 unit Taq polymerase (Qiagen, Hot Star Master Mix, Hilden, Germany). All amplifications were performed in a Peltier Thermal Cycler (PTC-0200 version 4.0, MJ Research) under the following conditions: an initial denaturation at 95°C for 15 min, followed by 35 cycles of 1 min at 94°C, a 45 s annealing step at 53°C, a 1 min extension step at 72°C, with a final 10 min at 72°C. The PCR products were cloned into a pCR2.1 vector using the TOPO TA cloning^®^ kit (Invitrogen, Carlsbad, CA, United States). The inserts were amplified with colony PCR using M13 forward and reverse primers, and the sequencing reaction was carried out with the Big Dye^®^ Terminator v3.1. cycle sequencing kit (Applied Biosystems, Darmstadt, Germany). Sequencing reactions were run on an ABI 3100 genetic analyzer (Applied Biosystems). Three to six different clones of the fragments were sequenced using both M13 forward and M13 reverse primers in order to identify the presence of different haplotypes within individuals (heterozygotes) and to control for Taq polymerase sequencing errors. The cloned and sequenced fragments were identified with a TBLASTX search, in order to analyze their structural features (exons and introns) and their nucleotide diversity in the different oak samples. In case of TPS1 and TPS6 the sequences were divided into two fragments since the overlap of 10 bp between the sequences generated by the forward and reverse primers was not sufficient.

### Analysis of Sequences

The allelic variation of the terpene synthase genes was analyzed by comparative sequencing of the samples used in the herbivory experiment and additional eight individuals of each population. Thus, populations “R45” and “BB1” were each represented by up to 12 individuals and each individual was represented by two haplotypes (Table 1). For editing and visual inspection of the sequences, as well as for the analysis of single nucleotide polymorphisms (SNPs) and indels (insertions/deletions) within the gene, the sequences were aligned with Codon Code Aligner (Codon- Code cooperation)^4^ and BioEdit version 7.0.0 (Hall, 1999) using clustalw multiple alignment (Thompson et al., 1994). Only polymorphisms with Phred scores above 25 in the chromatograms were considered. For estimating the standard population genetic parameters (e.g., number of segregating sites S, nucleotide diversity p, population differentiation F_ST_), DnaSP 5 (Rozas et al., 2003) was used. The number of Single Nucleotide Polymorphisms (SNPs), nucleotide diversity (π) and allelic variation measured as haplotype diversity (Hd) are summarized in Table 1. Tajima’s *D* neutrality test was used in order to find alleles or SNPs under selection. Negative values are usually interpreted as a sign of negative or purifying selection, while positive values are a signature of positive, balancing or diversifying selection. Singletons were excluded from all analyses.

**Table 1:** Allelic variation of the terpene synthase genes in the populations “BB1” and “R45.”

Gene	*N*	bp	SNP	*h*	Hd	π	Tajima’s *D*
*TPS*1 fr1	46	527	30	21	0.951	0.0106	-0.672
*TPS*1 fr2	46	611	30	22	0.961	0.0081	-0.922
*TPS*6 fr1	46	640	72	36	0.986	0.0220	-0.523
*TPS*6 fr2	46	592	25	19	0.929	0.0070	-0.890
*TPS*7 fr1	44	626	65	37	0.993	0.0318	1.161
*TPS*12	46	1062	50	36	0.989	0.0128	0.674
*TPS*13	42	1114	45	31	0.976	0.0062	-1.202


*N, sample size; bp, B = length in base pairs; SNP, number of SNPs (without singletons); h, number of haplotypes; Hd, haplotype-diversity; π, nucleotide diversity (per site); Tajima’s *D*, neutrality test.*

### Gene Expression Analysis by Quantitative Real-Time Polymerase Chain Reaction

RNA was extracted from roots (*N* = 6) of infested and non-infested plants of populations “BB1” and “R45” according to Le Provost et al. (2007). RNA (15 mg) from each sample was treated with DNase (RNasefree) at 37°C for 30 min to eliminate contamination with genomic DNA, then extracted with phenol/CHCl_3_ (1:1) and CHCl_3_ and precipitated overnight at -80°C with NaAc 1:10 and 2.5 volumes of absolute ethanol. cDNA was synthesized from 1 μg of the treated RNA samples using oligo(dT) primers and Superscript III reverse transcriptase (Invitrogen) at 50°C overnight. Based on the genomic sequences of the terpene synthase genes and of Actin, primers for quantitative real-time PCR were designed within the exon regions of the identified genes (Supplementary Table S3) using primer 3 (see above) and checked by PCR with genomic DNA as template. Quantitative real-time PCR was carried out on a LightCycler 480 (Roche) and monitored with SYBR-Green I dye (Roche). Melting curve analysis was performed for each primer pair by default and did not indicate off-target sequencing. Triplicates of 10 μl PCR reactions of each sample were done. Relative expression levels were analyzed using the ΔΔcp method (Livak, 1997, 2001; Livak and Schmittgen, 2001). The relative expression of the terpene synthase genes was normalized with the expression of the reference gene Actin in order to compensate for inter-PCR variations between the runs.

### VOC Analyses

To identify the VOCs released from oak roots (herbivory and bioassay experiments), we used self-made passive samplers as originally described by Kallenbach et al. (2014) which were furthermore tested particularly for their suitability for soil systems (Eilers et al., 2015). For this purpose, PDMS tubing (inner diameter 1.0 mm, outer diameter 1.8 mm, Roth, Germany) was cut into 5 mm long pieces and subsequently washed exactly as given by Kallenbach et al. (2014). For conditioning, passive samplers were placed in empty thermodesorption tubes (Gerstel, Germany) which were heated to 200°C under a N_2_ flux of 30–40 ml min^-1^ (N_2_ 5.0, Messer, Germany) in a Gerstel tube conditioner unit (Gerstel, Germany) for 6 h.

To test the suitability of such passive samplers for our purposes, a preliminary experiment was conducted in which the recovery rates of the passive samplers were characterized. We placed a piece of quartz wool spiked with authentic standards of the monoterpene (R)-(+)-limonene (123 nmol), the oxygenated monoterpenoids linalool (111 nmol) and the sesquiterpene β-farnesene (79 nmol) in the center of the same pots (*n* = 4) as used for plant experiments. Passive samplers were placed in a circle around this VOC source. After 7 days of exposure, the passive samplers were removed from the soil and analyzed as described above. It became evident, that all terpenes present in the source were detectable with our system (Supplementary Figure S2a). However, this experiment also indicated that the efficiency to trap a compound strongly depends on its chemical properties. In a second approach, we tested the linearity of trapping different amounts of volatiles. For this purpose, passive samplers were placed in solutions containing different amounts of limonene, linalool and farnesene. Importantly, we observed a linear correlation between the amount of the compound present in the solution and the compound trapped on the passive sampler (Supplementary Figure S2b).

The volatiles adsorbed on the passive samplers were analyzed by GC-MS (GC 6890, MS 5975C, both Agilent Technologies, Waldbronn, Germany) equipped with a thermodesorption unit (TDU, Gerstel, Müllheim an der Ruhr, Germany) coupled with a cold injection system (CIS, Gerstel, Müllheim an der Ruhr, Germany) (Eilers et al., 2015) For this purpose, the thermodesorption tubes containing passive samplers were inserted into the TDU by a Multipurpose Sampler (MPS 2, Gerstel, Müllheim an der Ruhr, Germany). After thermodesorption of analytes at 220°C for 4 min, analytes were cryofocussed in the CIS at -50°C. After heating up the CIS to 240°C for 3 min, analytes were flushed onto the separation column (DB-5MS Ui, 30 m, 0.25 mm ID, 250 μm, Agilent Technologies, Waldbronn, Germany). Details of GC and MS settings are provided by Kleiber et al. (2017). Raw data of GC-MS analysis were further processed by the MassHunter Quantitation software (Agilent, Waldbronn, Germany). Calibration was realized by using terpene standards, which were solved in hexane at different concentrations ranging from 0 to 20 nmol μl^-1^.

### Statistical Analysis

All statistical analyses were performed using Origin 2018G (OriginLab Corporation, Northampton, MA, United States). For the comparison of two groups the non-parametric Mann–Whitney *U* test was conducted (with significance level from *p* < 0.01–0.001) and to check more than two groups against each other we used the non-parametric Kruskal–Wallis ANOVA (*p* < 0.05). Gene expression and volatile data were log transformed before they were further analyzed. Differences between the volatile pattern released by roots of different origins were highlighted using a multivariate partial least squares discriminant analysis (PLS-DA) in MetaboAnalyst 4.0^5^ (Xia et al., 2015).

In order to calculate putative associations between genetic difference and infestation strength, we performed a Mantel test in GenAlEx (Peakall and Smouse, 2006). To do so, a dissimilarity matrix was created between the 14 *Q. robur* populations. Infestation strength was defined by a yes/no decision of infestation absent (0) and infestation present (1), corresponding to a low/not relevant and high cockchafer population density, respectively (Supplementary Table S1). When performing the Mantel test, cockchafer condition was correlated with unbiased Nei’s genetic distances using 999 permutations.

## Results

### Population Genetic Analysis With Nuclear and Chloroplast SSRs

The genetic characterization of the studied populations based on the nSSRs showed a clear differentiation between the different species *Q. robur, Q. petraea, Q. pubescens* present at the different field sites (Supplementary Figure S3). Importantly, for further studies only pure-bred *Q. robur* individuals were selected.

The molecular variation among the 14 *Q. robur* populations was relatively low (2%, data not shown) and no strong differentiation between the provenances was observed if tested by PCA (Supplementary Figure S4). Only populations “BB2” and “R36” differed from the others (Supplementary Figure S4). Also, no correlation between geographic and genetic distance was observed (Mantel test, *R*^2^= 0.0277). Similarly, no association between Nei’s unbiased genetic distance and the presence of infestation could be detected in a Mantel test (*R*^2^ = 0.0014).

In accordance with the results reported by Neophytou et al. (2010), all investigated nuclear SSRs were highly polymorphic with a total number of alleles ranging from 9.09 in “SA4” to 18.91 in “R36” (Supplementary Table S4). High allelic diversity and heterozygosity were observed in our samples. Overall unbiased expected heterozygosity varied between 0.802 in population “BB2” and 0.831 in population “BY1.”

**FIGURE 1:**
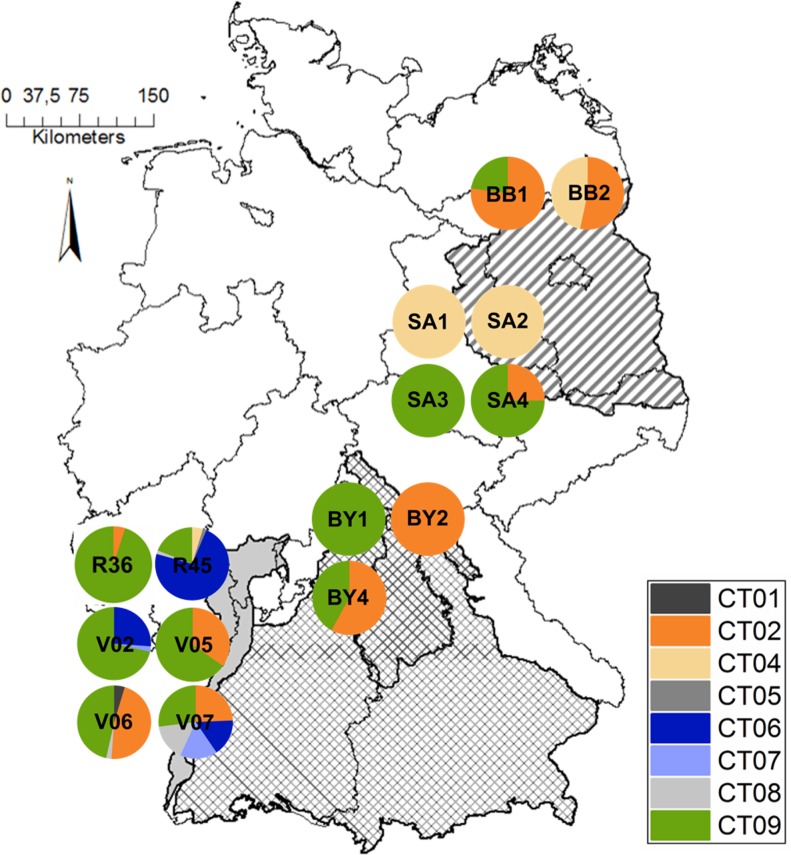
Distribution of found chlorotypes (CT) in *Q. robur* individuals within sampled populations in Baden-Württemberg (R36, R45, V02, V05, V06, V07), Bavaria (BY1, BY2, BY4), Saxony-Anhalt (SA1, SA2, SA3, SA4) and Brandenburg (BB1, BB2). The three relevant German *Q. robur* provenances ‘Oberrheingraben’ (81707, gray uni), ‘Süddeutsches Hügel- und Bergland sowie Alpen’ (81709, checkered) and ‘Ostdeutsches Tiefland’ (81704, lined) are highlighted. The map of Germany is based on a map from Esri Germany (https://opendata-esri-de.opendata.arcgis.com/).

Chloroplast analyses were performed to test for autochthony of stands, since different susceptibility to cockchafer infestation could be associated with different geographic origins. All analyzed chloroplast microsatellite loci were variable possessing two to four alleles each, except for μkk3 which was monomorphic. In total, nine chlorotypes could be defined by combining the different genetic variants at each locus (Supplementary Table S5). For the purpose of comparability, chlorotypes were assigned to those described earlier (Petit et al., 2002; Neophytou and Michiels, 2013) (Supplementary Table S5). The most commonly found chlorotypes were chlorotypes 9 and 2, corresponding to chlorotype 9 (Lineage C, Italian) and chlorotype 6 (Lineage A, Balkan) as observed by Neophytou and Michiels (2013), respectively. Spatial distribution of the chlorotypes differed among populations, with those in the Upper Rhine Valley (Baden-Württemberg) being the most variable comprising chlorotypes from all lineages (Balkan, Iberian, Italian). Two populations in Bavaria (“BY1” and “BY2”) and three in Saxony-Anhalt showed only one chlorotype. While the Iberian lineage was absent from the populations of Bavaria, Brandenburg and Saxony-Anhalt, chlorotype 4 (Lineage A, Balkan) was present only in one population in Saxony-Anhalt (Figure 1). However, the analysis of the cpSSRs did not detect a correlation between Nei’s unbiased genetic distance and the presence of infestations in the populations (Mantel test, *R*^2^ = 0.0238).

### Oak Populations Are Differently Attractive for Cockchafer Grubs

**FIGURE 2:**
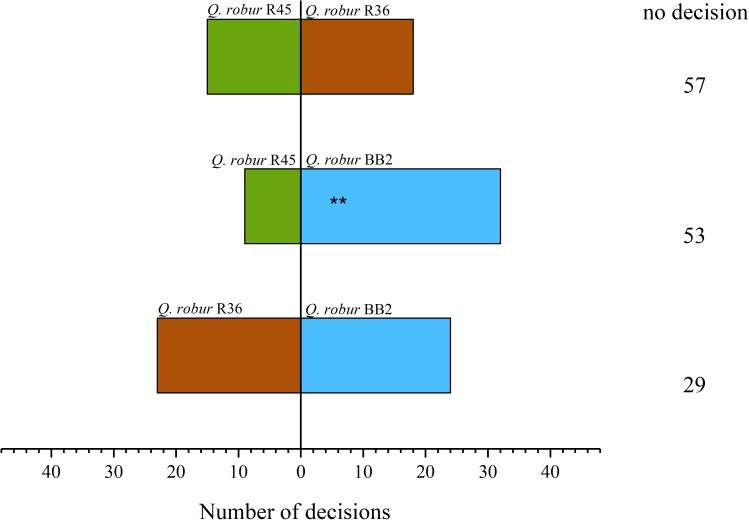
Olfactory bioassay with *M. hippocastani* larvae and three *Q. robur* populations. Two trees, one of two populations each, were tested against each other. Each of the three possible combinations was planted 10 times. Individual larvae were placed in the central inlet of the bioassay system for 12 h. Data are total numbers of animals selecting specific trees, or with no decision. Statistically significant differences were calculated with the Wilcoxon rank sum test (^∗^*p* < 0.05, ^∗∗^*p* < 0.01, ^∗∗∗^*p* < 0.001).

In an experiment under controlled conditions (“olfactory bioassay”), we tested if roots of the different oak populations from distant sites in Germany (Northeast Germany vs. Southwest Germany) are differently attractive to cockchafer larvae. The bioassays clearly indicated that trees from population “BB2” (NE Germany) were significantly more attractive to cockchafer larvae than trees from the SW German population “R45” (Figure 2). Interestingly, the reactions of the insects were comparable when the populations “R36” and “R45” from SW Germany were tested against each other in the bioassay. Similarly, there was no preference when “R36” was compared to “BB2.”

### Oak Populations Differ in Volatiles Released by Roots

**FIGURE 3:**
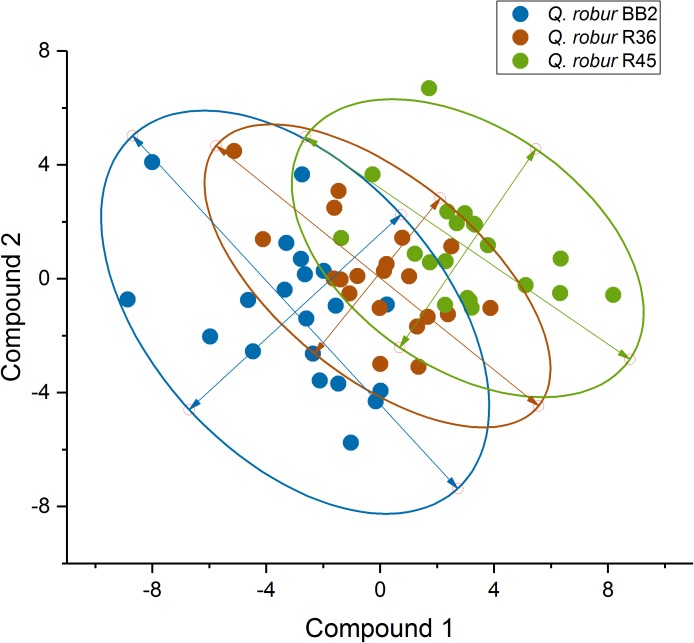
PLS-DA of the VOC patterns in the soil of three different *Q. robur* provenances (*N* = 20). The trees were kept for 3 months on the identical soil substrate and were used for the bioassays (see Figure 2). During the bioassays, volatiles abundant in the soil were collected on passive samplers and analyzed by GC-MS. Ellipses indicate the 95% confidence level.

**FIGURE 4:**
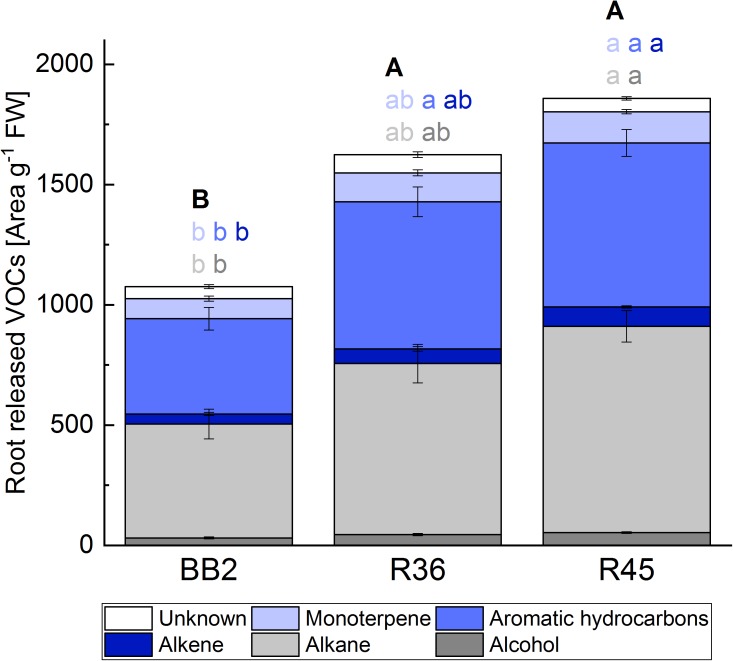
Root-released VOCs from trees of three *Q. robur* populations (*N* = 20). Data shown in Supplementary Table S6 were summarized according to main compound classes. Statistically significant differences between populations were calculated by Kruskal–Wallis-ANOVA (*p* < 0.05) for each compound class and are indicated by different letters (individual colures represent the different compound classes).

To test the hypothesis that different blends of root-released volatiles are responsible for the observed different preferences of the insects, the volatiles present in the soil of the trees were analyzed (Figure 3 and Supplementary Table S6). We observed that oak trees originating from different stands specifically affect the abundance of certain volatiles in their substrate. This became obvious when classifying the volatiles into different compound classes (Figure 4). Particularly, populations “R45” and “BB2” showed clear differences, whereas “R36” and “R45” did not greatly differ in their composition of volatiles. This pattern correlated with the preferences of the cockchafer larvae in the olfactory bioassay (Figure 2). Interestingly, many of the compounds differing in abundance between “BB2” and “R45” belonged to the classes of benzenoids and terpenes (Supplementary Table S6).

### Identification of Oak Terpene Synthase Genes and Their Structural Features

We amplified, cloned, sequenced and identified five different oak terpene synthase genes at the genomic level. They represent partial gene sequences and their structural features are described in Table 2. Following the nomenclature of Irmisch et al. (2014), *TPS*1 and *TPS*7 represent sesquiterpene synthases, whereas *TPS*6, *TPS*12 and *TPS*13 represent monoterpene synthases. At the genomic level they consisted of 1050–1450 bp, containing three to four introns and three to four exons (Table 2); they showed typical conserved elements like the DDxxD and the NSE/DTE motifs (data not shown). In genes *TPS*1, *TPS*6 and *TPS*12 also alternative splicing sites were found belonging to the class of U12 Introns (Lewandowska et al., 2004).

**Table 2:** Structural features of the identified oak terpene synthase genes in the populations “BB1” and “R45.”

	Intron	Exon 1	Intron	Exon 2	Intron	Exon 3	Intron	Exon4	Intron
*TPS* 1		1–192	193–321^∗^	322–417	418–596	597–845	846–946	947–1237	
*TPS* 6	1–37	38–250	251–338	339–716	717–933^∗^	934–1161	1162–1234	1235–1345	1346–1385
*TPS* 7	1–221	222–581	582–671	671–899	890–1225	1226–1363			
*TPS* 12		1–42	43–152	153–398	399–516	517–807	808–1055^∗^		
*TPS* 13		1–141	142–252	253–501	502–723	724–1026			


*^∗^Represents alternative splicing sites AT/AC instead of GT/AG.*

### Differentiation of the Terpene Synthase Genes Analyzed by Comparative Sequencing of Individuals of the Populations “BB1” and “R45”

In general, a high total nucleotide diversity (π) was found in all analyzed *TPS* genes ranging from 0.0062 to 0.0318, but no variation was found in the conserved motifs DDxxD and DxxDD. The genetic differentiation between populations, F_ST_, was calculated according to Wright (1951). Depending on the gene, a differentiation of up to 12.5% (*TPS*1) was found because some alleles were found in population “BB1” but not in population “R45.” However, *TPS*12 showed no differentiation between the two populations. Tajima’s *D* values did not differ significantly.

### Expression Rates of the Terpene Synthase Genes in Roots of Tree Samples of the Stands “BB1” and “R45” After Feeding

**FIGURE 5:**
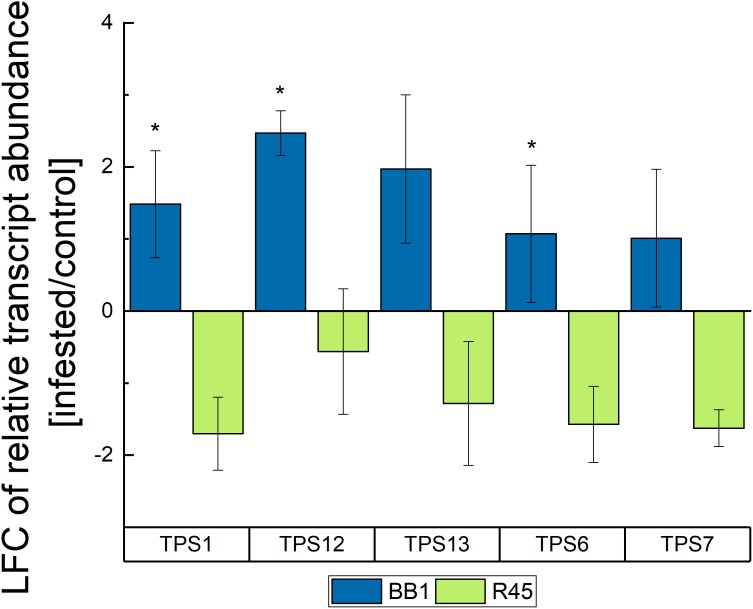
Effects of larvae-induced root damage on the expression of terpene synthase genes in roots (*N* = 6) of trees of two *Q. robur* populations (“BB1” and “R45”). Displayed are the means ± SE of the log2 values of the fold-changes (LFC) of relative gene expression between infested and control plants. Statistically significant differences between populations were calculated by Mann–Whitney *U* test for each TPS and are indicated by ^∗^*p* < 0.05.

In a controlled herbivory experiment, cockchafer grubs were allowed to feed on roots of oak seedling of different populations (“BB1,” “R45”). The RT-qPCR analysis showed distinct differences in the ΔCt values (Ct_TPS_ – Ct_house-keepinggene_) and therewith in the relative expression rate as analyzed as 2^-ΔΔCt^ for the identified TPS genes (Figure 5). In roots, while relative gene expression varied between individuals, the relative expression levels of all identified TPS genes generally increased in response to feeding. The expression ratio was generally higher in provenance “BB1” where upregulation after larval feeding was significant for *TPS*1, *TPS*6, and *TPS*12 (Figure 5).

### Herbivory Affects Terpene Blends

**FIGURE 6:**
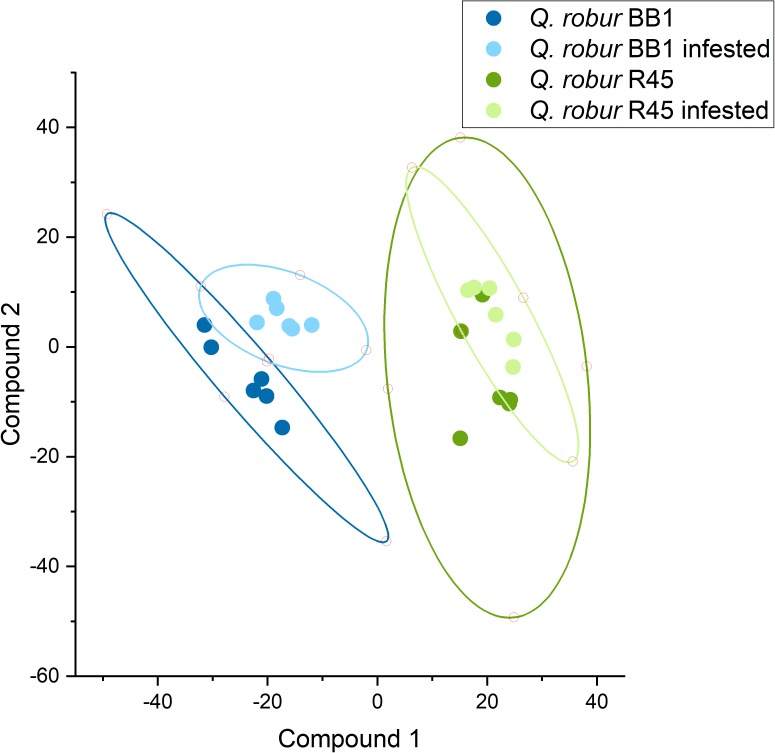
PLS-DA of root-released VOC patterns of two different *Q. robur* populations (*N* = 6). Six trees of each population were infested for 7 days by two cockchafer grubs each. Volatiles in the soil were trapped by passive samplers which were placed in the soil during the course of the experiment. Ellipses reflect the 95% confidence levels.

We investigated the effect of root herbivory on the release of VOCs from the roots of the non-infested site “BB1” in NE Germany and “R45” from SW Germany, which revealed the strongest differences in VOC release to the control trees (Figures 3, 4) and the largest differences in the interaction with cockchafer larvae in the bioassay (Figures 2, 6 and Supplementary Table S7). Trees of both populations showed changes in the release of VOCs from roots after infestation by cockchafer grubs. The differences between non-infested controls and cockchafer-infested plants were most prominent in population “BB1” where many compounds showed an altered pattern of VOC released into the soil (Figure 6). Among the compounds with increased levels in response to root herbivory were several benzenoids but also many compounds of other chemical classes (Supplementary Table S7). Roots of population “R45” also responded to exposure to cockchafer larvae but the effects were less pronounced than the reactions of “BB1.”

## Discussion

In the present study, we performed experiments under controlled conditions in which we tested the attraction of soil-dwelling cockchafer larvae by the root system of *Q. robur* trees from different populations. We compared trees from non-infested sites of NE Germany (populations “BB1” and “BB2”) with trees from a region in SW Germany where the forest cockchafer in general is quite abundant (populations “R36” and “R45”). However, despite their proximity, stand “R36” is highly infested, whereas stand “R45” is far less infested by cockchafer larvae as assessed by a long-term monitoring program (Delb and Mattes, 2001; Delb, 2004). Therefore, the question arises if these differences are caused by environmental factors or rather by plant internal factors such as the genetic constitution resulting in different physiological responses.

When compared in the olfactory bioassays, the cockchafer larvae preferentially selected trees from NE Germany (“BB2”) compared to population “R45” from SW Germany. This result was unexpected because of the low natural abundance of the cockchafer in stand “BB2” but the general high presence of this insect in SW Germany. We therefore speculate that oak trees from SW Germany evolved some kind of adaptation to the occurrence of the forest cockchafer in this region including, e.g., a repellent effect of the roots mediated by root-released volatiles. Since the forest cockchafer is not present in NE Germany, such a defense mechanism against the larvae might not have evolved in local oak provenances.

Due to the design of our bioassays, only volatile signals released by the root systems of the experimental trees could have determined the choice of the animals. The olfactory detection system of cockchafer larvae has been studied intensively and it was demonstrated that the chemosensory capacities of the larvae of this species are well developed (Eilers et al., 2012). Electroantennograms and electropalpograms indicated that cockchafer larvae are able to perceive a broad range of volatiles including alcohols, acids, amines, esters, aldehydes, ketones and monoterpenes (Eilers et al., 2012). Such compounds can be attractive or repellent to soil-dwelling insects (Johnson and Gregory, 2006). Movement toward a tree of population “BB2” in the present experiments can therefore be caused by perception of an attractant released by the root system or a repellent from the opposing tree (i.e., a tree of population “R45”). Considering the low natural rates of infestation at stand “R45” despite the high abundance of the forest cockchafer in this region, we hypothesize that “R45” releases trace gasses into the soil which act as repellents, most likely contributing to the constitutive defense against herbivore attacks. This assumption is in accordance with the observed distinct differences in the volatile scent released by roots of the three *Q. robur* populations (Figure 3 and Supplementary Table S6). In good agreement with the results of the bioassays, populations “R45” and “BB2” showed the strongest differences in the abundance of volatile compounds in the soil, whereas only small differences were identified between the two SW German populations on the one hand and between “R36” and “BB2” on the other hand.

The volatile compounds released by the root system can provide hints for such assumed repellent properties. The very low levels of VOCs in the soil of “BB2” might indicate the absence of repellent compounds, leading to the preferential choice by cockchafer larvae in the bioassays. Notably, trees of population “R45” in general released much higher amounts of a large variety of different compounds such as aromatics, terpenes as well as alkenes and alkanes than trees of “BB2” (Figure 4). Among the aromatics, we identified several compounds which have been discussed to be involved in plant defense. For example, the benzenoid benzothiazole, which was more abundant in the soil of “R45” than of “BB2,” seems to be released during induced defense in response to wounding (Bruin et al., 1992; Ping et al., 2001; Mithöfer et al., 2005; Hu et al., 2009) and was suggested as a volatile signal mediating plant–plant communication (Hu et al., 2009). Similarly, benzyl alcohol which is involved in defense against biotic stress (Widhalm and Dudareva, 2015; Szewczyk et al., 2016; Wang et al., 2016) was more abundant in the soil of “R45” than in “BB2.” Deterrent effects against herbivores have also been ascribed to members of the chemical class of terpenes (War et al., 2012). Among the 7 terpenoids identified in the soil of the trees (α-pinene, camphene, δ-3-carene, limonene, sabinene, *p*-cymene, isomenthol; Supplementary Table S6), the contents of δ-3-carene, the aromatic monoterpenoid *p*-cymene and the oxygenated compound isomenthol were significantly higher in the soil of “R45” than of “BB2.” Eilers et al. (2012) demonstrated that from the compounds present in the soil of our oak trees, at least α-pinene, limonene and camphene can be perceived by the cockchafer larvae. The monoterpenes α-pinene and camphene, which did not show significant differences in abundance between “BB2” and “R45,” exerted attractive effects on larvae of the common cockchafer (Eilers, 2012). Unfortunately, information on the perception and effects of δ-3-carene, *p*-cymene and isomenthol is lacking, and should be in the focus of future work.

It is well understood that herbivory induces a plethora of plant defense mechanisms at different levels including numerous plant secondary compounds (Walling, 2000; War et al., 2012). Induced defense often includes the release of herbivore-induced plant volatiles (HIPVs) (Robert et al., 2012). HIPVs are non-polar, volatile compounds which are released either into the atmosphere mainly from leaves, or into the soil from the roots in response to herbivore attack (Dudareva et al., 2006, 2013). These gasses can directly impair the animal’s vitality, act as feeding deterrent, or indirectly attract predators of the herbivore in tritrophic interactions (Farag and Paré, 2002; Dudareva et al., 2006; Arimura et al., 2009; Dicke and Baldwin, 2010; War et al., 2012; Kaling et al., 2018). As important part of a plant’s defense arsenal against herbivore attack we therefore aimed at clarifying if *Q. robur* trees of different populations differ in their response to infestation by cockchafer larvae. Because of the clear differences between the NE German population “BB2” and the SW German population “R45” in the bioassays (Figure 2) and in the release of volatiles from the roots system (Figures 3, 4), we included two populations from these origins in the analysis on the effects of root herbivory. Due to the lack of trees from “BB2,” in this experiment we had to work with “BB1” which originates in close vicinity to “BB2” with assumed very similar properties. Indeed, trees of population “BB1” showed clear effects on the VOC release after 8 days of cockchafer infestation whereas trees of “R45” did not show such strong reactions as indicated by multivariate analysis (Figure 6). Interestingly, the release of some volatile compounds decreased in response to exposure to the herbivore. In “BB1” this was most pronounced for undecyl-cyclopentane, isopropyl myristate, heptadecane and propanetrial triacetate. In contrast, roots of “R45” released lower amounts of mainly β-pinene, trimethylbenzene, and cyclododecane in response to herbivory (Supplementary Table S7). There is not much information on these compounds in the literature, although emission of some of them has been reported for other plants. Isopropyl myristate, trimethylbenzene, and heptadecane, for example, are emitted by several plant species, but their functions are not known yet (Thompson and Gornall, 1995; Weissteiner, 2010; Yang et al., 2014; Martins et al., 2017). According to the electrophysiological studies of Eilers et al. (2012), the common cockchafer is not able to perceive β-pinene so that lower emission of this compound cannot be related to the interaction with this herbivore, supposing that the forest cockchafer used in the present study has similar capabilities.

However, we also observed elevated abundance of several compounds in the soil of infested trees of “BB1.” It was obvious that the abundance of some aromatic compounds strongly increased. Several studies reported that herbivory on aboveground plant parts activates the jasmonic acid-induced phenylpropanoid pathway, presenting an important defense reaction of plants against biotic stress (Kazan and Manners, 2008; Rasmann and Agrawal, 2008; Lanoue et al., 2010). The present study provides hints that similar processes also occur in belowground parts of oak trees. The observed reactions in *Q. robur* roots might therefore be interpreted as an induced stress defense response in this tree species. Accordingly, some of the compounds showing elevated abundance have been reported to fulfill defense functions in other species. As mentioned above, benzenoids such as benzaldehyde, benzyl alcohol, and benzothiazole most likely play a role in defense against biotic stress (Bruin et al., 1992; Ping et al., 2001; Mithöfer et al., 2005; Hu et al., 2009; Widhalm and Dudareva, 2015; Szewczyk et al., 2016; Wang et al., 2016). Similarly, 1,2,3-trimethylbenzene was abundant in approximately 2-fold higher amounts in the soil of infested “BB1” compared to controls. This result is consistent with increased emission rates of this volatile from tropical plants infested by leaf herbivores (Martins et al., 2017). In good agreement with our results, these authors also observed higher release of the aldehydes nonanal and decanal, which therefore also seem to be involved in biotic stress defense. Moreover, in our study the abundance of the alcohol 2-ethyl-1-hexanol was significantly increased in the soil of infested trees of population “BB1.” This compound has been shown to be released from leaves of the tea plant in response to treatment with the plant hormone methyl jasmonate which acts as an efficient elicitor of secondary metabolite production under biotic stress (Shi et al., 2015). The emission of similar compounds from the roots of apple trees was also induced by the forest cockchafer (Abraham et al., 2015). Consistent with the work of Eilers et al. (2012), we also observed increased emission of 2-ethyl-1-hexanol in oak roots damaged by cockchafer larvae. In addition, Eilers et al. (2012) observed enhanced emissions of 1-octen-3-ol, anisol, octan-3-one and the oxygenated monoterpene eucalyptol in response to infestation of oak roots. However, none of these compounds were detected in our study which might be caused by the different sampling techniques used in both studies. Such compounds might have been adsorbed by soil particles and, thus, did not reach our passive samplers (Ramirez et al., 2010) or the efficiency of the samplers was too low for such compounds. Since our test experiments indicated that a broad range of compounds with different polarities was efficiently trapped by the samplers, the former assumption seems to be more likely.

Among the compounds abundant in higher amounts in the soil of infested trees were also some terpenes (Supplementary Table S7). Increased emission of terpenes has often been observed in herbivore-infested plants (Unsicker et al., 2009; Dudareva et al., 2013; Martins et al., 2017). Terpene release is assumed to protect the roots by their antimicrobial and antiherbivore properties. In tritrophic interactions, they can also attract natural enemies of the root feeding insect pests. For example, the sesquiterpene β-caryophyllene which is synthesized in corn roots due to infestation by the larvae of *Diabrotica virgifera* attracts the nematode *H. megidi* (Rasmann et al., 2005). However, sesquiterpenes were not detected in the soil during our studies which might be due to the short half-life time of these compounds. Nevertheless, strong hints for stimulated production of mono- and sesquiterpenes is provided from our gene expression studies where an upregulation of several terpene synthases occurred in “BB1” in response to infestation which was not the case for “R45.” This finding is in good agreement with an increased release of β-pinene and δ-3-carene from roots of infested of “BB1.” We propose that one of the assumed monoterpene synthases TPS13, TPS12, or TPS6 is responsible for the biosynthesis of these compounds. Other studies identified monoterpenes like myrcene (TPS13), terpinolene (TPS12 and TPS13), linalool (TPS12), and ocimene (TPS6) as the natural products of these genes. These terpene products have also been described as typical stress monoterpenes in green leaves of *Q. robur* after infestation with oak powdery mildew (Copolovici et al., 2014). The sesquiterpene synthases (TPS1 and TPS7) use farnesyl diphosphate (FPP) as substrate and the larger carbon skeleton of FPP increases the structural diversity of the products. In poplar TPS1 is known as germacrene D synthase and as a multiproduct enzyme (Eberl et al., 2017); also, TPS7 can produce more than 15 different sesquiterpenes with elemol being the main product (Irmisch et al., 2014). As mentioned above, we identified other terpenes in control trees of the different populations (Supplementary Table S6) which might be the products of the TPS of the *Q. robur* trees studied here. Therefore, one can assume that the allelic variation and also the alternative splicing sites found in three (*TPS*1, *TPS*6, and *TPS*12) of the identified TPS genes contribute to the terpene diversity. Similar results were also described by Huang et al. (2010) who analyzed the herbivore-induced volatile terpenes in different ecotypes of *Arabidopsis*. They also found that allelic diversification of TPS genes in wild gene pools contributes to the natural variation in terpene formation.

The genetic characterization of the studied populations regarding their origin as well as possible local adaptation processes permit a better understanding of their putative resilience to stresses, such as the herbivore effects of cockchafer larvae. Therefore, we characterized the genetic diversity of the plant material used in this study using nuclear and chloroplast SSRs. Those highly variable nuclear microsatellite loci are an effective tool for studying population genetic diversity, gene flow and genetic structure as well as for gathering information about adaptive processes (Neophytou et al., 2010). To date, they have been extensively used for studying the genetic properties of oak populations in European forests (Petit et al., 2002; Neophytou et al., 2010; Gerber et al., 2014). The main findings describe a higher genetic variation within than among oak populations (Kremer and Petit, 1993; Neophytou et al., 2010). Accordingly, the nuclear SSRs showed no distinct spatial genetic structuring of the studied populations. The same was true for the association between infestation strength and genetic properties where we could not detect a correlation between the presence of cockchafer infestation and Nei’s genetic distance at putatively neutral SSRs. The characterization of a large array of genes involved in defense against biotic stress is necessary to identify associations between genetic variation in genes and infestation strength in natural populations. The observed physiological differences in response to cockchafer feeding between the Brandenburg populations “BB1”/”BB2” and “R45” from Baden-Württemberg are most likely due to the absence of the forest cockchafer from Brandenburg so that defense mechanisms against the larvae have not evolved. In contrast, this cannot be an explanation for the differences in population density of cockchafer larvae of the neighboring stands “R45” and “R36.”

Contrarily, the chloroplast SSR analyses did reveal a distinct geographic structure of chlorotypes. This suggests a post-glacial recolonization of the studied populations by oaks originating from different refugial regions as described by Petit et al. (2002) (Figure 1). These results are in accordance with those stated by Neophytou and Michiels (2013), showing a high chlorotype variation in the Upper Rhine Valley and confirming the hypothesis of a merging point of the refugial migration routes from the West, the South and the Southeast. Consistently, the populations further east possess chlorotypes of the southeastern lineage (Balkan) in higher frequencies. In spite of their proximity, the two Baden-Württemberg populations “R45” and “R36” reveal a different composition of chlorotypes. The highly infested population “R36” is dominated by chlorotype 09 from the southern (Italian) lineage, whereas “R45” is dominated by chlorotype 06 from the western (Iberian) lineage (Figure 1).

There are studies showing that populations originating from different genetic lineages possess different local adaptation capacities (Anderson et al., 2011; Prunier et al., 2012; de Lafontaine et al., 2018). This might also be true for areas of high cockchafer population density such as the Upper Rhine Valley, where populations with chlorotype 09 show higher cockchafer infestation levels than populations with chlorotype 06. However, measured TPS gene expression and VOC emission were rather similar in populations “R36” and “R45.” Therefore, we assume that other genes are involved in the defense mechanisms against cockchafer infestation or other factors such as specific habitat characteristics regulate cockchafer infestation on those sites. But this remains to be confirmed by more detailed studies.

## Conclusion

The present study has demonstrated that *Q. robur* trees from spatially distant populations with differing genetic background induce different reactions in soil dwelling forest cockchafer larvae. Surprisingly, trees originating from regions with low abundance of this herbivore species were more attractive to the insect larvae than trees from SW Germany where cockchafer density is high. Olfactory bioassays clearly suggested that the difference in insect attraction was caused by different patterns of root-released VOCs. Since the SW German population released higher amounts of VOCs into the soil, we assume a repellent effect against cockchafer larvae of at least some of these compounds. The presence of several benzenoids and terpenes, both groups of chemicals known to be involved in plant defense, support this view. Interestingly, we also observed that the release of such compounds was stimulated by herbivory, underlining their role in plant defense against root feeding insects. As seen from gene expression studies as well as from the analysis of root abundant volatiles, the oak populations obviously differed in the production of HIPVs. This finding suggests that plant defense against herbivory is population specific and therefore under genetic control.

Different strengths of infestation with the cockchafer of closely neighboring stands (“R45,” “R36”) can have several reasons. Root related processes such as differences in root attraction as affected by the release of plant volatiles as well as differences in plant defense after wounding by a herbivore can play a role only at a small spatial scale. However, at larger scale the selection of oviposition sites is of greater importance. Location of suitable feeding and oviposition sites by host-seeking insects might be mediated by volatile chemical cues (Webster and Cardé, 2017). Such volatiles are released from habitats in greater amounts than from single host plants and are detectable by the insects over longer distances (Webster and Cardé, 2017). Future studies should clarify if volatile chemical cues released from different oak forest stands differ in composition and, consequently, influence the selection of oviposition and feeding sites of the forest cockchafer.

## Author Contributions

FC performed the sampling, carried out the GC-MS analysis, evaluated the data, and wrote the manuscript. IC performed the sampling and the genotyping. HD, AD, OG, and LL were involved in writing the manuscript. JK performed data analysis and wrote the manuscript. JT-S and BV performed the sampling, genetic characterization, data analysis, and wrote the manuscript.

## Conflict of Interest Statement

The authors declare that the research was conducted in the absence of any commercial or financial relationships that could be construed as a potential conflict of interest.

## Footnotes

1 www.meyer-shop.com

2 http://primer3.ut.ee/

3 http://www.basic.northwestern.edu/biotools/oligocalc.html

4 https://www.codoncode.com

5 http://www.metaboanalyst.ca
