# Supplementary figures and images for: Terpene Synthase Genes in Quercus robur – Gene Characterization, Expression and Resulting Terpenes Due to Cockchafer Feeding
Source: Front Plant Sci. 2018 Nov 30;9:1753. doi: 10.3389/fpls.2018.01753 (PMC6287202; doi:10.3389/fpls.2018.01753)

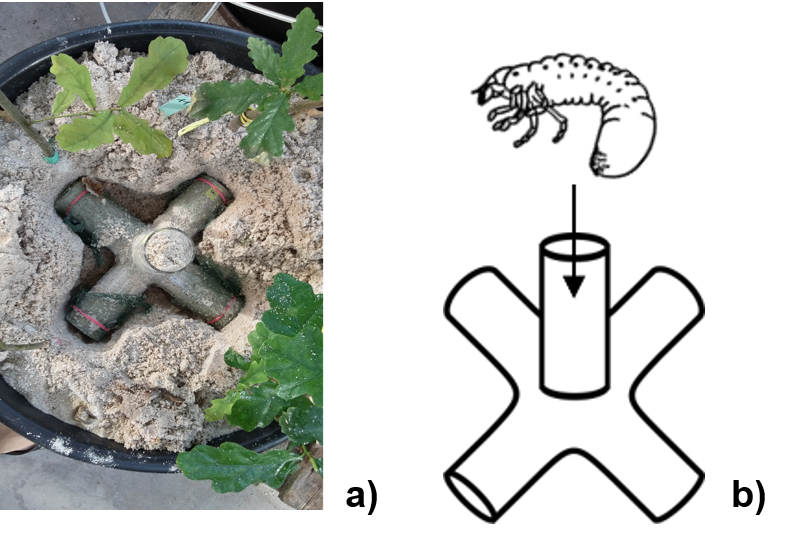

Supplement: Supplementary file 1 [file Image_1.TIF]

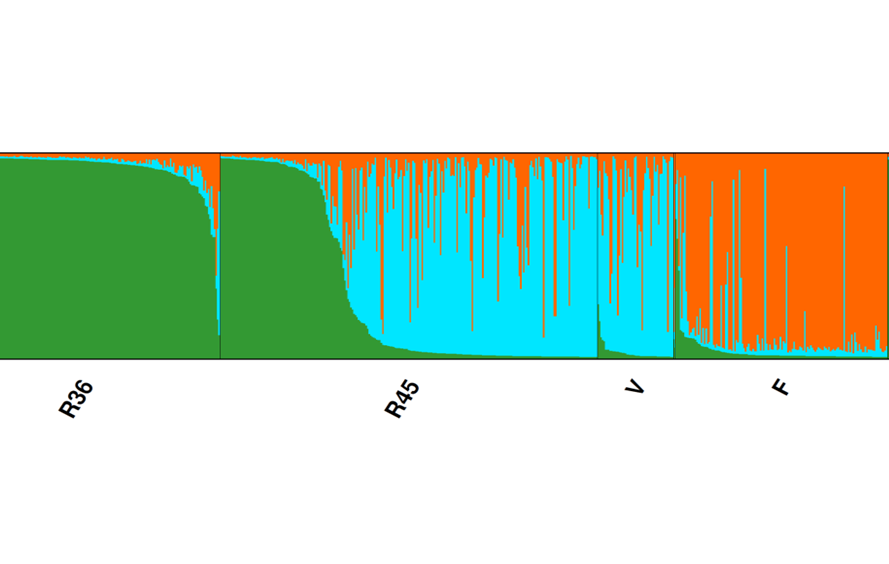

Supplement: Supplementary file 3 [file Image_3.TIFF]
