# Supplementary material for: Terpene Synthase Genes in Quercus robur – Gene Characterization, Expression and Resulting Terpenes Due to Cockchafer Feeding
Source: Front Plant Sci. 2018 Nov 30;9:1753. doi: 10.3389/fpls.2018.01753 (PMC6287202; doi:10.3389/fpls.2018.01753)
Supplement: Supplementary file 5 [file Table_1.pdf]

Supplemetal Tables

Supplemental Table S1 - Summary of characterized oak populations. Climate data by DWD, German Weather Service, range from 1986 to 2016.

Population	DG N	DG E	Species	Cockchafer population density	Federal State	Provenience	Average annual precipi-tation [mm]	Average annual temper-ature [°C]	Average temperature Oct-March [°C]	Average temperature Apr-Sept [°C]
V	47.79425	7.64908	*Q. petraea*	NR	BW	817 07	930.6	10.4	8.0	12.8
BW	47.81340	7.65904	*Q. petraea, Q. pubescens*	NR	BW	817 07	930.6	10.4	8.0	12.8
F	48.06991	7.60751	*Q. pubescens*	NR	BW	817 07	687.8	11.2	5.5	16.6
R45	49.10011	8.42247	*Q. robur, Q. petraea*	low	BW	817 07	689.7	11.2	5.4	17.0
V02	49.20073	8.54688	*Q. robur*	high	BW	817 07	689.7	11.2	5.4	17.0
R36	49.21734	8.56104	*Q. robur*	high	BW	817 07	689.7	11.2	5.4	17.0
V05	49.27788	8.64979	*Q. robur*	high	BW	817 07	689.7	11.2	5.4	17.0
V06	49.27945	8.65054	*Q. robur*	high	BW	817 07	689.7	11.2	5.4	17.0
V07	49.34357	8.60767	*Q. robur*	low	BW	817 07	689.7	11.2	5.4	17.0
BY1	49.68093	10.20322	*Q. robur, Q. petraea*	NR	BY	818 09	600.5	10.0	4.2	15.9
BY4	49.75361	10.18830	*Q. robur*	NR	BY	820 09	600.5	10.0	4.2	15.9
BY3	49.85169	10.15062	*Q. petraea*	NR	BY	821 09	583.1	11.1	4.1	15.7
BY2	49.88252	10.26084	*Q. robur,*	NR	BY	819 09	806.4	8.4	2.9	13.9

			Q. petraea							
SA2	52.05124	12.04616	*Q. robur*	NR	SA	819 04	567.5	9.7	3.7	15.5
SA1	52.05235	12.03641	*Q. robur, Q. petraea*	NR	SA	820 04	567.5	9.7	3.7	15.5
SA4	52.08819	12.30665	*Q. robur, Q. petraea*	NR	SA	821 04	567.5	9.7	3.7	15.5
SA3	52.09933	12.28916	*Q. robur*	NR	SA	822 04	567.5	9.7	3.7	15.5
BB2	52.44405	13.09461	*Q. robur*	NR	BB	818 04	588.5	9.5	3.7	15.4
BB1	52.77092	13.15959	*Q. robur*	NR	BB	818 04	522.8	9.7	3.9	15.5

NR, not relevant; BW, Baden-Württemberg, BY, Bavaria, SA, Saxony-Anhalt, BB, Brandenburg

Supplemental Table S2 - Summary of the BLASTx search of the translated terpene synthase (TPS) nucleotide sequences within the NCBI BLAST+ database. For each TPS, poplar access number, the percentage of identity with the poplar sequence and the corresponding E value is mentioned. For the amplification and identification of the different TPS genes at genomic level, DNA of one individual of population R36 was used.

	poplar access number	percentage of identity to poplar sequence and corresponding E value at nucleotide level (including introns)	at protein level (only exons)
***TPS*1**	JF449450	82 %, 1e-146	73 %, 5e-125

```
oak       FEQKRGHVASAVECYMAQHGGTEEEAINEFCKQVTDAWKDINKECLYPTTVPMPILMRIL
          FEQKRGHVAS++ECYM QHG TE+E ++EF KQVTDAWKD+N+E L+PT VPMP+L R+L
poplar    FEQKRGHVASSIECYMKQHGTTEQETVHEFRKQVTDAWKDVNEEFLHPTAVPMPLLTRML

oak       NLARVIDVVYKDEDGYTHAGIVLKDFVTLMFVDPVP
          NLARVIDVVYKDEDGYT+AG  LKD V+ + +DPVP
poplar    NLARVIDVVYKDEDGYTNAGTALKDLVSALLIDPVP
```

	poplar access number	at nucleotide level (including introns)	at protein level (only exons)
***TPS*6**	KF776504	74 %, 1e-100	78 %, 2e-133

```
oak       INNKDTKLLTTLELIDDIERLGLGYRFKEEIMRALDRFVTLKGCEEFTNGSIHDTALSFR
          INN+  + LT LELID+++RLGLGYRF+ +I RALDRFV+  G +  T  S+H TALSFR
poplar    INNEKAEFLTLLELIDNVQRLGLGYRFESDIRRALDRFVSSGGFDAVTKTSLHATALSFR

oak       LLRQHGFGVSQDMFNCFKDQKGNFKECLSKDIKGLLSLYEASYLGFEGENLLDEAREFTT
          LLRQHGF VSQ+ F  FKDQ G F E L +DIK +LSLYEAS+L  EGEN+LDEA+ F
poplar    LLRQHGFEVSQEAFGGFKDQNGKFLENLKEDIKAILSLYEASFLALEGENILDEAKVFAI

oak       MHLKDLKGD-VSRTLKEEVRHSLEMPLHRRMRRLEQRWYIDAYNMKEAHDRKLLELAKLD
           HLK+L  + + + L E+V H+LE+PLHRR +RLE  W I+AY  KE  ++ LLELA LD
poplar    SHLKELSEEKIGKDLAEQVNHALELPLHRRTQRLEAVWSIEAYRKKEDANQVLLELAILD
```

oak	FNIVQSVHQRDLKDMSRYLRWWQEMGLGNKLSFARDRLMECFFFSVGMAFEPQFSNSRKA		
	+N++QSV+QRDL++ S RWW+ +GL KL FARDRL+E F+++VG+AFEPQ+S+ R +		
poplar	YNMIQSVYQRDLRETS---RWWRRVGLATKLHFARDRLIESFYWAVGVAFEPQYSDCRNS		
oak	VTKMFSFITVIDDIYDVYATLEELEMFTDIVQRFRWDVKAVKDLPEYVKLCFLALFNTVN		
	V KMFSF+T+IDDIYDVY TL+ELE+FTD V+ RWDV A+ DLP+Y+KLCFLAL+NT+N		
poplar	VAKMFSFVTIIDDIYDVYGTLDELELFTDAVE--RWDVNAINDLPDYMKLCFLALYNTIN		
oak	EMVYDTLKEQG		
	E+ YD LKE+G		
poplar	EIAYDNLKEKG		
**_TPS_7**	KF776505	74 %, 1e-114	69 %, 1e-102
oak	KVKD--GQFKESLASNVKGMLAFYEATHLRVHGEGILDEALEFTTTHLKSTVSAICNPLA		
	K KD G FK+S ++V+G+L YEA HL VHGE ILDEAL FTT HLKS S+ PL		
poplar	KFKDDKGYFKQS--NDVRGILGLYEAAHLAVHGEDILDEALAFTTIHLKSMASSPNCPLT		
oak	EQVTRALKQPLHKGIPRLEARQYISIYKQDASHNKVFLMLAKLDFDMVQSLHKEELSYIT		
	+V+ ALKQP+ +G+PRLE+R+YISIY+ + S NK L LAKL+F++VQ LHKEEL+ IT		
poplar	AKVSHALKQPIQRGVPRLESRRYISIYQDEPSCNKTLLRLAKLNFNLVQELHKEELAEIT		
oak	RYSRWWKDLDFVKKLPFARDRVVEGYXGIVAVYXEPQYSXARKILTKVIAMTSIIDDIYD		
	RWWK LDF ++LPFARDRVVE + IV VY EPQYS ARKILTKVIAMTSIIDDIYD		
poplar	---RWWKGLDFARRLPFARDRVVECFFWIVGVYFEPQYSLARKILTKVIAMTSIIDDIYD		
oak	VYGTLEELEPFTEAIERF--		
	VYGTLEELE FTEAI+R+		
poplar	VYGTLEELELFTEAIDRWDTKSMDQLPDYMKICYEALLNVFSEIEEKVAKEGWSYRVHYG		

```
oak      ---MKLLVRAYFDEAKWFHQNYIPTMEEYMNLALRTSGYPMLTTVSFLG
            MK+LV AYF+EAKWFH+N+IPTMEEYM +AL TSGY MLTTVSF+G
poplar   KDAMKVLVHAYFNEAKWFHENHIPTMEEYMQVALVTSGYSMLTTVSFIG
```

***TPS*12**	KF776510	85 %, 6e-64	73 %, 1e-70

```
oak      LKNQWANLCRAYLLEAKWYYNGYTPSLQEYLDNAWVSVSVPTILLNTYFSATNPITKEAL
         LK  WA+LC++YLLEAKWY++GYTP+LQEY+DNAW+S+S P IL++ YF  +NP T+EA
poplar   LKKAWADLCKSYLLEAKWYFSGYTPTLQEYMDNAWISISAPVILVHAYFYVSNPTTEEAS

oak      DFF--------WSSMIIRLVDDLGTS----ERGDVPKSIQCYMNETGASEEDAYEYIRCL
          F         WSSMI+RL DDLGTS    +RGD+ KSIQCYM+E G SEE A E+IR L
poplar   QFMEEYPDIIRWSSMILRLADDLGTSTDELKRGDISKSIQCYMHEAGVSEEKAREHIRNL

oak      ISAIWKKINEERAETSPFSDTFIEIIFNIVRVAHCMYQYGDGHGVGNHETKDCLLSLFVQPI
         I   WKKIN+ + +    S TFI I  N+ R+A CMYQYGDGHGVG+ ETKD + SL ++P+
poplar   IENTWKKINDYQFDNPRISQTFIGIAMNLARMAQCMYQYGDGHGVGHLETKDRVKSLLIKPL
```

***TPS*13**	KF776511	85 %, 7e-111	85 %, 4e-116

```
oak      RWDINAIDELPDYMKICFIALHNSINEMTFDTLKEQGFHVIRFFKKAWADICRSYLLEAK
         RWD+N +D LPDYMK+CF+AL NS+NEM ++ LK QG  ++ + KKAWAD+C+SYLLEAK
poplar   RWDLNYMDRLPDYMKLCFLALFNSVNEMAYNILKYQGVDILPYLKKAWADLCKSYLLEAK

oak      WYHSGYTPSLQEYIENAWISISAPTILVHAYFFVTNPITKEGLDCLEEYPNIIRWSSMIL
         WY SGYTP+LQEY++NAWISISAP ILVHAYF+V+NP T+E    +EEYP+IIRWSSMIL
poplar   WYFSGYTPTLQEYMDNAWISISAPVILVHAYFYVSNPTTEEASQFMEEYPDIIRWSSMIL
```

```
oak      RLADDLGTSTDELKRGDV-PSIQCYMNETGASEEEAREHIRFLISATWKKMNEDRAASSP
         RLADDLGTSTDELKRGD+  SIQCYM+E G SEE+AREHIR LI  TWKK+N+ +  +
poplar   RLADDLGTSTDELKRGDISKSIQCYMHEAGVSEEKAREHIRNLIENTWKKINDYQFDNPR

oak      FSETFIEIALNLARMAQCMYQHGDGHGAGNHETKDRVLSLLIQPI
          S+TFI IA+NLARMAQCMYQ+GDGHG G+ ETKDRV SLLI+P+
poplar   ISQTFIGIAMNLARMAQCMYQYGDGHGVGHLETKDRVKSLLIKPL
```

Supplemental Table S3 – Cloning primer sequences of terpene synthase genes.

	PCR		qRT-PCR	
**Terpene-synthase* (poplar) EMBL Access.No**	**Orthologous++ sequence in *Q.robur***	**Primer sequence (5' to 3')**	**Primer sequence (5' to 3')**	**Amplified length in exon region (bp)**
TPS 1<br>JF449450	gnl|OCV4_assembly_final|OCV4_rep_c36581	F: TTG GAC TTG TCG AGG AAG CTA**<br>R: GGC ACA GGA TCA ATA AGC ATC **	F: GGG AGT GTA CTT TGA GCC AGA<br>R: GCT CAA CCT CTT CAA GTG TGC	119 (exon 1)
TPS 6<br>KF776504	gnl|OCV4_assembly_final|Loc_86506	F: AAA GAA GCT GGG GGA AGA AG<br>R: CCC CAC GCT TTT GTT AGG TA	F: TGA GCT TTG CCA GAG ACA GA<br>R: CAG TTC TTC CAA GGT GGC ATA	161 (exon 2)
TPS 7<br>KF776505	gnl|OCV4_assembly_final|OCV4_rep_c16869	F: AAT GTT GCC CTT TG TTT TCG<br>R: CCA AGG AAA GAC ACG GTT GT	F: GAG CCC CAA TAC TCA CTT GC<br>R: TCA GTG AAG GGC TCA AGT TC	116 (exon 1)
TPS 12<br>KF776510	gnl|OCV4_assembly_final|Loc_11416	F: TCC AAC CGA AGT TCA CAA TG<br>R: AGG AAC AAG GAT TCC ACA TCA	F: TGC ATG GGT ATC AGT GTC AG<br>R: TTA TGT TGG GGC ATT CTT CG	110 (exon 1)
TPS 13<br>KF776511	gnl|OCV4_assembly_final|Loc_24254	F: ATC CAA AGG AAA AGG GGA AA<br>R: TTCC AGA TGG GAT ATC AAT GC	F: CGG ATA TAC ACC GAG CCT TC<br>R: AAC AAT CCA AGG CCT CCT TT	125 (exon 2)
Actin			F: AAG GCC AAC AGG GAA AAG AT<br>R: GTC ACC AGA GTC CAG CAC AA	135

*the nomenclature followed Irmisch et al.2014
** according to Kersten et al. 2013
++ according to oak transcriptome database available at: https://urgi.versailles.inra.fr/blast/blast.php.

Supplemental Table S4 - Genetic variation within populations calculated over all loci and considering only the *Q. robur* individuals. Number of samples (N), number of different alleles (N_a), number of effective alleles (N_e), unbiased expected heterozygosity (uH_e), observed (H_o) and expected heterozygosity (H_e), Fixation Index (F). Means ± SE, standard error.

Pop	N	Na	Ne	Ho	He	uHe	F
**R36**	163.91 ± 1.11	18.91 ± 2.06	6.99 ± 1.05	0.76 ± 0.04	0.81 ± 0.04	0.82 ± 0.04	0.07 ± 0.04
**R45**	68.64 ± 0.28	17.64 ± 1.84	7.42 ± 1.12	0.76 ± 0.05	0.8 ± 0.05	0.81 ± 0.05	0.04 ± 0.02
**V02**	43.27 ± 0.3	17.09 ± 2.05	8.72 ± 1.46	0.75 ± 0.05	0.82 ± 0.05	0.83 ± 0.05	0.08 ± 0.03
**V05**	49.64 ± 0.15	13.36 ± 1.3	6.56 ± 0.76	0.77 ± 0.04	0.81 ± 0.03	0.82 ± 0.03	0.04 ± 0.05
**V06**	42.45 ± 0.31	16.64 ± 1.54	8.14 ± 1.21	0.73 ± 0.05	0.81 ± 0.06	0.82 ± 0.06	0.09 ± 0.03
**V07**	37.73 ± 0.14	15.64 ± 1.84	7.08 ± 1.07	0.74 ± 0.05	0.8 ± 0.05	0.81 ± 0.05	0.06 ± 0.03
**BY1**	23 ± 0.4	13.91 ± 1.54	8.01 ± 1.23	0.77 ± 0.05	0.81 ± 0.05	0.83 ± 0.05	0.05 ± 0.05
**BY4**	32.27 ± 0.27	15 ± 1.84	8.4 ± 1.44	0.75 ± 0.05	0.81 ± 0.05	0.82 ± 0.05	0.07 ± 0.04
**SA1**	33 ± 0.5	13.64 ± 1.5	7.49 ± 1.11	0.82 ± 0.05	0.81 ± 0.04	0.82 ± 0.04	-0.02 ± 0.03
**SA2**	42.91 ± 0.39	15.09 ± 1.32	7.36 ± 1	0.76 ± 0.06	0.8 ± 0.05	0.81 ± 0.05	0.04 ± 0.05
**SA3**	36.64 ± 0.2	14.64 ± 1.66	6.96 ± 1.1	0.75 ± 0.05	0.79 ± 0.05	0.8 ± 0.05	0.05 ± 0.04
**SA4**	11.45 ± 0.28	9.09 ± 1.1	6.6 ± 0.87	0.78 ± 0.07	0.77 ± 0.07	0.81 ± 0.07	-0.02 ± 0.04
**BB1**	34.45 ± 0.21	14.55 ± 1.69	7.63 ± 1.08	0.72 ± 0.06	0.79 ± 0.06	0.81 ± 0.06	0.08 ± 0.04

**BB2**	30.55 ± 0.65	12 ± 1.2	6.11 ± 0.94	0.74 ± 0.06	0.79 ± 0.04	0.8 ± 0.04	0.07 ± 0.06
**Total**	**46.42 ± 2.82**	**14.8 ± 0.46**	**7.39 ± 0.29**	**0.76 ± 0.01**	**0.8 ± 0.01**	**0.82 ± 0.01**	**0.05 ± 0.01**

Supplemental Table S5 - Chlorotypes found in the study populations, considering only the *Q. robur* individuals, and assignment to chlorotypes defined by previous studies (Petit et al., 2002; Neophytou and Michiels, 2013).

Chlorotype	ccmp2	ccmp6	ccmp10	µcd4	µcd5	µdt1	µdt3	µdt4	µkk3	µkk4	Corresponding haplotype from Neophytou et al. (2013)	Corresponding refugial population
**1**	234	101	112	94	78	80	123	144	102	115	4	-
**2**	234	101	112	94	78	80	124	144	102	115	6	Balkan (A)
**4**	234	101	112	95	78	79	124	144	102	115	8	Balkan (A)
**5**	234	101	111	95	79	81	123	143	102	115	3	-
**6**	235	101	111	95	79	81	123	143	102	115	12	Iberian (B)
**7**	235	102	111	95	79	81	123	143	102	115	15	Iberian (B)
**8**	235	101	111	95	79	82	123	143	102	115	13	Iberian (B)
**9**	234	101	112	95	78	80	123	145	102	116	9	Apennin (C)

Supplemental Table S6: Root-released BVOC from three *Q. robur* populations (n = 20 each) used in a bioassay experiment. The peak areas of each treatment are shown as means ± SE. Statistically significant differences between the populations were identified by nonparametric Kruskall-Wallis-ANOVA ($P < 0.05$) and are marked by different letters.

Compound	CAS number	KI	Match [%]	BB2	Population R36	R45
Benzene, ethyl-	100-41-4	869	98.7	49 ± 7 **b**	73 ± 8 **ab**	83 ± 7 **a**
1,2-Dimethylbenzene (o-xylene)	0-00-0	872	98.9	170 ± 24 **b**	252 ± 29 **a**	286 ± 25 **a**
α-Pinene	80-56-8	932		30 ± 4	43 ± 5	42 ± 4
Camphene	79-92-5	953		2 ± 1	2 ± 0	1 ± 0
Benzene, 1-ethyl-3-methyl-	620-14-4	960	95.6	74 ± 10 **b**	109 ± 13 **ab**	116 ± 10 **a**
Sabinene	3387-41-5	970		3 ± 0 **b**	4 ± 1 **ab**	5 ± 0 **a**
Benzene, 1,2,3-trimethyl-	526-73-8	987	98.6	65 ± 8 **b**	93 ± 10 **ab**	97 ± 7 **a**
Octanal	124-13-0	1001	93.0	4 ± 0 **b**	6 ± 1 **a**	7 ± 1 **a**
δ-3-Carene	13466-78-9	1004		19 ± 3 **b**	31 ± 4 **ab**	34 ± 3 **a**
1,2,4-Trimethylbenzene (ϕ-cumene)	95-36-3	1017	94.2	31 ± 4 **b**	44 ± 5 **ab**	49 ± 4 **a**
Limonene	138-86-3	1025		10 ± 2	11 ± 1	12 ± 1
Benzyl alcohol	100-51-6	1039	96.9	29 ± 6	53 ± 12	44 ± 9
Benzene, 1-ethyl-2,4-dimethyl-	874-41-9	1052	86.5	15 ± 2 **b**	25 ± 3 **ab**	30 ± 2 **a**
Nonane, 4-methyl-5-propyl-	62185-55-1	1052	86.7	32 ± 5 **b**	50 ± 6 **ab**	60 ± 4 **a**
Ethanone, 1-phenyl-	98-86-2	1062	95.2	44 ± 8 **a**	66 ± 9 **ab**	90 ± 22 **b**
p-cymene	99-87-6	1083		18 ± 2 **b**	29 ± 4 **ab**	36 ± 3 **a**
3-Hexene, 3,4-dideutero-2,2,5,5-tetramethyl	22808-06-6	1091	80.3	13 ± 2 **b**	18 ± 3 **ab**	26 ± 4 **a**
1-Heptanol, 2-propyl-	10042-59-8	1099	82.5	25 ± 3 **b**	37 ± 4 **a**	43 ± 3 **a**
Nonanal	124-19-6	1099	94.3	91 ± 11 **b**	124 ± 12 **ab**	161 ± 19 **a**
Decane, 2-methyl-	6975-98-0	1113	85.6	117 ± 21 **b**	167 ± 22 **ab**	218 ± 18 **a**
1-Tridecanol	112-70-9	1114	84.1	126 ± 20 **b**	172 ± 21 **ab**	215 ± 18 **a**
1-Octanol, 2-butyl-	3913-02-8	1118	86.5	13 ± 2 **b**	21 ± 3 **ab**	26 ± 2 **a**
1-Methylpentyl cyclopropane	6976-28-9	1127	83.2	34 ± 5 **b**	44 ± 5 **ab**	52 ± 5 **a**
1-Dodecanol, 2-methyl-, (S)-	57289-26-6	1127	83.6	8 ± 3	8 ± 2	3 ± 0

Octadecane, 1-chloro-	3386-33-2	1150	84.7	8 ± 1 **b**	14 ± 2 **ab**	18 ± 2 **a**
Tridecane, 6-methyl-	13287-21-3	1150	83.8	40 ± 5 **b**	60 ± 7 **ab**	70 ± 6 **a**
Undecane, 5-methyl-	1632-70-8	1158	87.8	34 ± 5 **b**	54 ± 7 **ab**	65 ± 6 **a**
Decane, 3-methyl-	13151-34-3	1158	89.1	35 ± 5 **b**	55 ± 7 **ab**	65 ± 6 **a**
Nonadecane	629-92-5	1167	83.6	25 ± 3 **b**	40 ± 5 **a**	46 ± 4 **a**
Decane, 1,1'-oxybis-	2456-28-2	1171	81.0	25 ± 4	39 ± 6	34 ± 5
(+)-Isomenthol	23283-97-8	1178	89.3	6 ± 1 **b**	8 ± 1 **ab**	10 ± 1 **a**
Naphthalene	91-20-3	1182	95.7	190 ± 23 **b**	308 ± 40 **ab**	379 ± 41 **a**
Dodecane	112-40-3	1196	87.7	49 ± 7 **b**	69 ± 8 **ab**	90 ± 9 **a**
n-Decanal	112-31-2	1206	94.6	14 ± 2 **b**	22 ± 2 **a**	29 ± 4 **a**
Undecane, 2,6-dimethyl-	17301-23-4	1217	87.1	34 ± 4 **b**	45 ± 5 **ab**	52 ± 4 **a**
Tetradecane	629-59-4	1217	83.0	14 ± 2 **b**	22 ± 3 **ab**	25 ± 2 **a**
Benzothiazole	95-16-9	1219	88.0	28 ± 3 **b**	50 ± 8 **a**	60 ± 19 **ab**
Cyclohexane, 2-butyl-1,1,3-trimethyl-	54676-39-0	1242	81.5	25 ± 6	30 ± 8	41 ± 7
Dodecane, 2,6,11-trimethyl-	31295-56-4	1313	84.2	13 ± 2	15 ± 2	16 ± 1
Trans-3-hexanal	69112-21-6	1345	93.0	7 ± 1 **b**	20 ± 4 **a**	22 ± 6 **ab**
Benzene, 1,4-dichloro-2-nitro-	89-61-2	1357	95.7	5 ± 1	11 ± 2	10 ± 2
Phenol, 2-chloro-4-(1,1-dimethylpropyl)-	5323-65-9	1415	84.8	67 ± 12	94 ± 15	102 ± 13
Acetamide, N-methyl-N-[4-[4-methoxy-1-hexahydropyridyl]-2-butynyl]-		1438	85.6	11 ± 2	13 ± 2	18 ± 3
2,5-Cyclohexadiene-1,4-dione, 2,6-bis(1,1-dimethylethyl)-	719-22-2	1458	92.9	120 ± 14	191 ± 31	192 ± 36
9-Octadecenoic acid (Z)-	112-80-1	1493	82.4	7 ± 1 **b**	11 ± 1 **a**	10 ± 1 **a**
Propanoic acid, 2-methyl-, 1-(1,1-dimethylethyl)-2-methyl-1,3-propanediyl ester	74381-40-1	1585	82.9	140 ± 15 **b**	252 ± 49 **ab**	206 ± 19 **a**
Hexadecane	544-76-3	1607	87.7	15 ± 2 **b**	25 ± 3 **a**	26 ± 3 **a**
1,2-Benzenedicarboxylic acid, bis(2-methoxyethyl) ester	117-82-8	1867	86.2	43 ± 6 **b**	96 ± 17 **a**	104 ± 22 **a**
Hexadecanoic acid, methyl ester	112-39-0	1927	81.1	32 ± 7	47 ± 9	58 ± 20

Supplemental Table S7: The root systems of trees from two *Q. robur* populations (“BB1” and “R45”) were exposed to two *M. hippocastani* larvae for eight days. Released root VOCs from infested and control plants (n = 6 each) were trapped (Kallenbach et al., 2014). The ratios of the normalized peak areas of infested vs. control trees were calculated (fold-changes, FC) and are indicated as their log2-values. Only compounds are shown with a log2 of at least +0.5 or -0.5. The color code indicates increased (blue) or decreased (red) levels under infestation.

Name	***CAS***	**KI**	**Match [%]**	**Log2 FC „BB1“**	**„R45“**
terpenoids					
β-Pinene	18172-67-3	940		2.51	-1.02
δ-3-Carene	13466-78-9	1001		1.33	-0.07
α-Pinene	80-56-8	938		-0.24	1.10
aromatics					
Benzylalcohol	100-51-6	1027	98.9	1.37	0.10
Benzaldehyde	100-52-7	960	96.4	1.30	-0.20
Benzene, 1,2,3-trimethyl-	526-73-8	987	91.2	1.10	-1.44
Ethanone, 1-phenyl-	98-86-2	1052	98.5	0.54	0.34
Benzenemethanol, .α.,.α.-dimethyl-	617-94-7	1075	87.3	0.17	0.88
Benzothiazole	95-16-9	1207	88.7	0.00	1.30
Benzaldehyde, 3,4-dimethyl-	5973-71-7	1204	85.6	-0.17	0.64
Benzeneethanol	60-12-8	1099	94.2	-0.17	0.53
others					
Hexadecanoic acid, methyl ester	112-39-0	1927	87.8	3.77	1.32
Tetracosane	646-31-1	2194	94.9	1.46	0.75
Ethanol, 2-(2-ethoxyethoxy)-	111-90-0	994	90	1.33	0.39
Ethanol, 2-butoxy-	111-76-2	926	92.7	1.05	0.03
2,5-Cyclohexadiene-1,4-dione, 2,6-bis(1,1-dimethylethyl)-	719-22-2	1446	93.3	0.93	0.00
n-Decanal	112-31-2	1194	99.1	0.84	0.06
1-Hexanol, 2-ethyl-	104-76-7	1020	93.6	0.77	-0.09
Cyclododecane	294-62-2	1464	93.8	0.72	-1.09
Naphthalene	91-20-3	1171	96.5	0.58	-0.35

Nonanal	124-19-6	1096	99.1	0.57	-0.32
dimethyl bis(3-iodopropyl)malonate	100166-57-2	1789	93.8	0.50	0.28
(Z)-5-(2-ethoxythoxy)-3-methyl-2-penten-1-ol	100605-19-4	1619	87.5	0.26	1.80
Heneicosane	629-94-7	2201	95.7	0.26	1.11
Octadecanal	638-66-4	1590	82.6	0.16	1.04
Hexane, 1-chloro-	544-10-5	868	98.8	-0.44	1.11
Octadecane	593-45-3	1798	95.3	-0.55	0.56
Nonadecane	629-92-5	1879	94.7	-0.67	0.76
1-Eicosanol	629-96-9	1686	84.6	-0.72	0.20
2-Furancarboxaldehyde	98-01-1	835	92.1	-0.78	0.72
Tricosane	638-67-5	1999	91.8	-0.92	0.93
1,2,3-Propanetriol, triacetate	102-76-1	1339	82.6	-1.09	0.21
Heptadecane	629-78-7	1686	91.9	-1.23	0.77
Isopropyl myristate	110-27-0	1827	85.3	-1.38	0.60
Cyclopentane, undecyl-	6785-23-5	1642	83.2	-1.79	0.43

$p < 0.05$ = *, $p < 0.01$ = **, $p < 0.001$ = ***; KI, Kovats retention index.

Supplemental Figures

Supplemental Figure S1: Experimental setup of the bioassay experiment. Three *Q. robur* provenances ("R36", "R45", "BB2") were planted in 15 pots. Two trees of two provenances were combined per one pot, while each of the three possible combinations was replicated five times. Self-made glass tubing systems (inner diameter 45 mm, length of each tube 100 mm) were placed in the center of the pots in a depth of 20 cm with the exits facing the root systems **(a)** and thereafter covered with soil substrate. One grub was placed carefully in the middle of the upper glass arm; after 12 h the location of the grub was determined **(b)**. For more details see materials and methods section.

Supplemental Figure S2: Test of the suitability of VOC passive samplers for our purposes; **(a)** the passive samplers were placed in planting pots filled with the plant substrate used in our experiments (N= 4). A source of the authentic terpene standards limonene, linalool and farnesene was placed in the center of the pots in a depth of 10 cm. After 7 days of exposure, the passive samplers were collected and terpenes analyzed by TD-GC-MS. The percentage of the original quantities are indicated above each bar. In a second approach **(b)**, increasing amounts of terpene standard mixtures (limonene, linalool, farnesene) were added to 400 µl m methanol (Sarstedt, Nuembrecht, Germany) in 1600 µl H_2O; three passive samplers were added. The samples were shaken at 30° C and 1400 rpm for 60 minutes. Afterwards the passive samplers were dried and analyzed as described in the materials and methods section.

Supplemental Figure S3: Species assignment by Bayesian cluster analysis in STRUCTURE. The inferred clusters correspond to the common oak species *Q. robur* (green), *Q. petraea* (blue) and *Q. pubescens* (orange). Each individual is represented by a vertical bar which is color-coded according to the determined membership proportions of the three clusters. "R36", "V" and "F" were used as reference populations for species assignment to *Q. robur*, *Q. petraea* and *Q. pubescens*, respectively. "R45" is an exemplary mixed stand (*Q. robur* and *Q. petraea*) in Baden-Württemberg with low cockchafer population density.

Supplemental Figure S4: Principal coordinates analysis of the sampled population using only *Q. robur* individuals. Populations are color-coded for Baden-Württemberg (green and brown), Bavaria (grey), Saxony-Anhalt (purple) and Brandenburg (blue).
